# Parental attitudes, beliefs, and practices related to firearm storage: a qualitative study

**DOI:** 10.1186/s40621-022-00400-x

**Published:** 2022-12-21

**Authors:** Christopher Schenck, Meghan Wilson, Gunjan Tiyyagura, Kirsten Bechtel

**Affiliations:** 1 Yale School of Medicine, New Haven, CT 06510 USA; 2 Department of Pediatrics, Yale School of Medicine, New Haven, CT 06520 USA; 3 Section of Emergency Medicine, Department of Pediatrics, Yale School of Medicine, New Haven, CT 06511 USA

**Keywords:** Pediatric firearm injury, Firearm storage, Injury prevention, Qualitative methods

## Abstract

**Background:**

Firearm injury is a leading cause of death among children. Safer firearm storage practices are associated with a reduced risk of childhood suicide and unintentional firearm death. However, these practices are underutilized. The objective of this study was to characterize parental attitudes and beliefs related to firearm storage and identify facilitators and barriers to safer storage practices.

**Methods:**

Semi-structured, qualitative interviews were conducted to identify motivations for using different storage methods among parents who kept firearms in southern Connecticut. The constant comparative method was used to code interview transcripts and derive themes directly from the data.

**Results:**

Twenty participants completed the study. 60% were male, 90% were white, and all were between 32 and 53 years old. 85% of participants stored firearms locked, 60% unloaded, 65% kept ammunition locked or did not keep ammunition in their home, and 45% stored ammunition separate from firearms. The following themes were identified: (1) firearm storage must be compatible with a specific context of use; (2) some parents engage in higher-risk storage because they believe it is adequate to reduce the risk of injury; (3) firearm practices are influenced by one’s social network and lived experience; (4) parents who own firearms may be amenable to changing storage practices; and (5) parents’ conceptualization of firearm injury prevention is multimodal, involving storage, education, and legislation.

**Conclusions:**

Parents who keep firearms value convenience and utility, which may be at odds with safer storage practices; however, some may be amenable to adopting safer practices. Family and peer relationships, education, and legislation represent important facilitators of storage practices. Understanding parental attitudes and beliefs on firearm storage may inform future interventions to improve storage practices.

## Background

Firearm injury is a leading cause of death among children younger than 18 years old in the USA. Almost 40% of firearm deaths in this age-group are suicides (WISQARS^TM^ 2022). Multiple studies have demonstrated a strong association between completed youth suicide and the presence of a firearm in the home (Brent et al. 1991, 1988, 1993; Shah et al. 2000). Additionally, states with higher firearm ownership have a higher rate of unintentional firearm deaths compared to states with low firearm ownership (Miller et al. 2001). While the presence of a firearm in the home is a primary risk factor for suicide or unintentional injury involving a firearm, the practices of locking firearms, storing firearms unloaded, locking ammunition, and storing ammunition separate from firearms reduce the odds of firearm suicide and unintentional injury (Grossman et al. 2005). The American Academy of Pediatrics recommends that all firearms are stored locked and unloaded, with ammunition locked separately (Dowd et al. 2012), which we will refer to as safer storage. Any other storage practice (e.g., keeping a firearm unlocked) will be referred to as higher-risk storage.

Despite the protective effect of storing firearms locked and unloaded, these practices are underutilized. Azrael and colleagues report that approximately one-fifth of firearm-containing households with children have at least one firearm kept unlocked and loaded, the highest risk storage method (Azrael et al. 2018). Those who keep firearms for self-defense, and those who keep handguns, are more likely to store firearms unlocked and loaded (Azrael et al. 2018). Johnson and colleagues found that homes with adolescents are more likely to contain unlocked firearms than those with younger children (Johnson et al. 2006), despite adolescents being at higher risk of firearm injury, such as suicide.

Understanding firearm owners’ attitudes and beliefs about firearm storage may elucidate reasons for the limited adoption of best practices. Many parents endorse firearm storage as important in preventing child firearm injury, while also emphasizing the importance of educating children to act safely around firearms (Barton and Kologi 2015; Howard 2005). Previous studies indicate that firearm owners may underestimate both the risk of keeping a firearm in the home and the risk-mitigating effect of safer firearm storage (Conner et al. 2018; Anestis et al. 2018; Connor and Wesolowski 2003; Farah et al. 1999). Fewer than 10% of parents who keep firearms report believing that firearms in the home increased the risk of suicide (Conner et al. 2018). Those who do not believe in an association between firearm storage and firearm suicide risk are more likely to store firearms unlocked (Anestis et al. 2018). Most parents also believe that their children would not touch a firearm if they found one (Connor and Wesolowski 2003; Farah et al. 1999). Parents also underestimate children’s knowledge of where firearms are stored and whether children have handled firearms (Baxley and Miller 2006).

Aitken and colleagues recently conducted a focus group study to understand beliefs regarding firearm storage among parents living in states with high firearm ownership (Aitken et al. 2020). The authors report that storing firearms locked and unloaded often conflicts with a perceived need to quickly access firearms to protect oneself, and parents also value educating children about safe firearm use (Aitken et al. 2020). Our qualitative study sought to further characterize why parents engage in different firearm storage behaviors, what sources parents use to inform decisions about firearm storage, and identify barriers to using safer storage practices.

## Methods

### Research team

KB and GT are pediatric emergency medicine physicians with expertise in qualitative research. CS is a medical student and MW is a senior pediatrics resident both trained in qualitative methods by KB.

### Setting and participants

The study was conducted between August 2019 and November 2020. Individuals were considered eligible for participation if they were the parents or guardians of a child less than 18 years old and lived in a home where a firearm was present. A convenience sample of parents who kept firearms was recruited from three general pediatrics clinics and a pediatric emergency department in New Haven County, Connecticut. Initially, recruitment was performed in-person by an investigator who screened potential participants for eligibility criteria. Due to COVID-19 procedures, in-person recruiting was terminated. After March 1, 2020, recruitment occurred via flyers distributed at recruitment locations with an invitation to contact the research team. Participants provided written consent if recruited in-person. Otherwise, they provided verbal consent via telephone. Participants received a $25 gift card for taking part in the study, and the research team offered a firearm cable lock to every participant. The institutional human research protection program of Yale University approved this study.

### Study design

Sociodemographic information and characteristics of firearms kept in the home were obtained using a standardized survey instrument. Individual semi-structured interviews were conducted either in-person or via telephone by CS, who is trained in qualitative interviewing. We chose to conduct individual interviews rather than focus groups to elicit more detailed information on decisions about firearm storage and to minimize the influence of conformance or censoring which may arise in focus groups (Kidd and Parshall 2000). The interview guide was developed by the authors with open-ended questions designed to elicit parents’ beliefs and practices related to firearm storage, and facilitators and barriers to safer storage. In accordance with grounded theory (Strauss and Corbin 1990), the interview guide was revised iteratively, with a minor modification clarifying Question 8 regarding motivations to change storage practices; the final version of the guide is presented in Table 1. All interviews were audio-recorded and were transcribed verbatim by a professional transcription service (*Landmark Associates Inc., Phoenix AZ*). At the onset of the study, interviews were conducted in person. However, due to the COVID-19 pandemic, all interviews after March 1, 2020, were conducted by telephone.

**Table 1:** Semi-structured interview guide

	1	Can you tell me about the benefits of keeping firearms for you and your family?		
	2	*If parent reports that their children use firearms—*Tell me more about how your children use the firearms in your home		
	3	How do you keep your children from getting injured by firearm(s) in your home?		
	4	Can you tell me more about the reasons for your practice?		
	5	How did you learn about these safety practices?		
	6	You said that you store your firearms (*storage method reported*). What makes this a good option for you?		
	7	What challenges or problems might there be with storing your firearms this way?		
	8	Have you ever considered changing how you stored your firearms?		
		a	*If participant considered change*: What made you consider this change? Did you change your storage practices?	
			i	*If participant considered change but did not change storage practices*: what kept you from changing your practices?
		b	*If participant did not consider change*: What would make you consider changing how you stored your firearms?	
	9	Many parents choose to store their firearms locked and unloaded in order to reduce their children’s risk of injury. What are your thoughts on this? (*if further prompting is needed:* what are the benefits and problems with storing your firearms this way?)		
		a	*If participant does not store all firearms unloaded and locked*: What keeps you from storing them this way?	
		b	What would get you to consider storing your firearms this way?	
	10	What do you think is the best way to keep children safe from getting injured by firearms? (*if further prompting is needed:* for example, laws that require firearms to be stored locked and unloaded, formal training held by law enforcement or sporting groups, informal instruction by peers, etc.)		
*If participant indicates that protection or self-defense is a reason for keeping firearms*				
	1	Have you ever worried about you or someone in your family being the victim of a crime?		
	2	Whose responsibility is it to keep you and your family safe?		
	3	What is your own role in keeping you and your family safe?		

These questions were used to guide conversation with each participant. Participants were free to make comments unrelated to these prompts, and the interviewer asked additional questions to further elaborate ideas expressed by participants

### Data analysis

KB, MW, and CS independently reviewed all interview transcripts and assigned descriptive codes. Codes were iteratively revised, combined, and organized using the constant comparative method (Hewitt-Taylor 2001). Discrepancies in coding were discussed until consensus was achieved. After the codebook was finalized, CS coded all transcripts with the final code structure. The research team met to group codes into categories and identify themes from the emerging data. Data collection ended when thematic saturation was achieved (Ando et al. 2014). Data management was performed using HyperRESEARCH qualitative data analysis software (*Researchware, Inc., Randolph, MA).*

## Results

Twenty participants completed the study (15 telephone interviews). Participants were majority male (60%), white (90%), and between 32 and 53 years old. Children present in the home had a mean age of 8 years. Sociodemographic data are presented in Table 2.

**Table 2:** Sociodemographic characteristics of study participants

	n (%)
Sex	
Female	8 (40)
Male	12 (60)
Age	
30–39	10 (50)
40–49	7 (35)
50 +	3 (15)
Race/ethnicity	
Non-Hispanic White	18 (90)
Non-Hispanic Black	2 (10)
Level of education	
Some college	4 (20)
College graduate	16 (80)
Household income	
$50,000–99,999	2 (10)
$100,000–149,999	7 (35)
$150,000 +	11 (55)
Marital status	
Single	2 (10)
Married	16 (80)
Divorced/separated	2 (10)
Children (age)	
0–4	14 (35)
5–9	8 (20)
10–14	13 (32.5)
15–17	5 (12.5)
Children (sex)	
Female	18 (45)
Male	22 (55)

Eighty-five percent of participants stored firearms locked, 60% stored firearms unloaded, 65% kept ammunition locked or did not keep ammunition in their home, and 45% stored ammunition separate from firearms. Complete data on the type and number of firearms and reasons for keeping firearms are presented in Table 3.

**Table 3:** Characteristics of firearm ownership and storage among study participants

	n (%)
Number of firearms	
1–5	13 (65)
6–10	4 (20)
> 10	3 (15)
Firearm type	
≥ 1 Handgun	17 (85)
≥ 1 Rifle	12 (60)
≥ 1 Shotgun	8 (40)
Firearm storage	
Locked and unloaded	10 (50)
Locked and loaded	7 (35)
Unlocked and unloaded	2 (10)
Unlocked and loaded	1 (5)
Ammunition storage	
Locked and separate	2 (10)
Locked and not separate	9 (45)
Unlocked and separate	5 (25)
Unlocked and not separate	2 (10)
No ammunition	2 (10)
Reason for ownership	
Target/trap/skeet shooting	14 (70)
Protection of self and others	12 (60)
Hunting	5 (25)
Inherited/gift	3 (15)
No personal use	3 (15)
Collecting	1 (5)
Dealing/trading	1 (5)
Child knowledge/access	
Knows firearm is in home	15 (75)
Knows where firearm is kept	9 (45)
Able to access firearm	2 (10)
Uses firearm	0 (0)

Firearm and ammunition storage are reported as the least secure method if multiple methods are employed, e.g., a participant who keeps 1 firearm loaded but others unloaded would be coded as ‘loaded.’ Ammunition storage is coded as ‘separate’ if it is kept in a location separate from all firearms. Participants could indicate multiple reasons for ownership. Firearms that were kept unlocked were coded as accessible to children

The five main themes identified are presented below. Selected quotations representative of these themes are presented in Table 4.

**Table 4:** Themes related to parental firearm storage practices

Theme	Representative Quotations
Firearm storage must be compatible with a specific context of use	*I do not need a side arm for protection in my home, and therefore it’s very easy for me to basically break this thing [the firearm] down into … less hazardous parts, but … there are people who keep handguns more readily available*
	*[quotation from participant who kept firearm for self-defense] It’s timing, because literally the safe that’s next to my bed is within my arm’s reach*
Some parents engage in higher-risk storage because they believe it is adequate to reduce risk of injury	*[quotation from participant who kept an unlocked, unloaded firearm] I felt that it was safe enough … Is it perfect? No, certainly could we put a gun safe some place in the house, I guess*
	*Not owning one is probably the best way [to keep children safe from injury] … If you're going to have them in the house, I think a specifically designed safe … is the next best thing*
Firearm practices are influenced by one’s social network and lived experience	*Safe practice, informing your children, begets safe practice. That’s how my father was. He was very responsible. I shot weapons from the time I was six years old*
	*I did eight years in the Marine Corps and I pretty much carry on that, the safety aspect for the firearms in my personal life*
Parents who own firearms owners may be amenable to changing storage practices	*When I was 21, I didn’t have children … My pistols were in the drawer, which weren’t locked … But when I started living with—me and [my partner], we came together, and then we had our daughter, [and] it was like, “Okay, things have gotta change. They don’t need to be on display … They need to be safe.”*
	*She [my wife] goes, “You know I wouldn’t mind if you bought another safe to keep all the ammunition separate.” I was like, you know, that’s actually not an awful idea now that I think about it*
	*Currently, there's no purpose for it. We don't use it. It's like, get rid of it*
Parents’ conceptualization of firearm injury prevention is multimodal, involving safer storage, education, and legislation	*Well, storing them locked is the primary means of ensuring safety, right? If you can’t access the firearms, you cannot use them. Then having them … unloaded is just a double lock and key-type measure. It’s kind of redundant*
	*She knows at six years old her basic rules of gun safety, which is … If you see a gun, run away and get an adult*
	*Controlling who can have them [firearms] with better background check situations*

Themes were derived through iterative coding of interview transcripts. Representative quotations of the identified themes are provided

### Theme 1: firearm storage must be compatible with a specific context of use

Eight of 12 participants who reported keeping firearms for self-defense cited timing and accessibility as key considerations in firearm storage. Some attributed keeping firearms loaded and/or unlocked to ensure quick access: “if someone's in your house, you have literally seconds before they're right there in your face. So, you have to find the key, get to the box, then you got to get to the ammo, unlock it, put it all together, I'm already dead at that point.” Some also reported that the location of the firearm was selected to ensure accessibility.

### Theme 2: some parents engage in higher-risk storage because they believe it is adequate to reduce risk of injury

Seven participants acknowledged that their current storage practices were not maximally safe but were adequate for their specific circumstances. The reasons endorsed for not using the safest possible storage practices varied. Some stated that young children were less likely to access or be able to use firearms. Three participants acknowledged that not keeping any firearms would be best for child safety, but still made the decision to keep firearms within the home. One stated, “Well, not owning one is probably the best way [to keep children safe from firearm injury.].”

### Theme 3: firearm practices are influenced by one’s social network and lived experience

Thirteen participants had family members that kept firearms and many participants grew up in households with firearms. Family members who kept firearms were important sources of information on firearm storage and safety for participants. Friends who kept firearms were also sources of information on firearm safety: “I have some friends that I go to the range with and one of them does have young kids. He is very adamant that you store the bullets and the firearm separately.” Some cited experience in military or law enforcement, or education from a family with experience in military or law enforcement.

### Theme 4: parents who own firearms may be amenable to changing storage practices

Eight participants considered adopting safer storage practices at the time of the interview. Four planned to use safer storage practices when their young children became older, due to the belief that older children were more likely to find a firearm: “Our 2-year-old is not capable now of finding [the firearm] … getting into a lock box and all of that.” Two participants considered removing firearms from the home. Three participants stated that discussing firearm injury prevention during the interview prompted consideration of adopting safer practices: one wanted to remove firearms from the home, one wanted to lock a previously unlocked firearm, and the other wanted to keep ammunition separate from firearms.

Four participants reported having previously improved firearm safety practices. Three stated having children prompted this change.

### Theme 5: parents’ conceptualization of firearm injury prevention is multimodal, involving safer storage, education, and legislation

Participants identified multiple factors that they believed were important for firearm injury prevention. Storing firearms locked, unloaded, and storing ammunition locked and separate from firearms were identified by participants as practices intended to prevent child injury. Several participants cited the prevention of firearm theft as an additional benefit of safer storage practices: “You worry about somebody being able to steal it and use it for another purpose.”

Firearms education for both adults and children was almost universally identified as important in preventing child firearm injury. There was substantial heterogeneity in desired content and setting. Some preferred instruction that emphasized the danger of firearms and strictly avoided firearms: “Don’t touch guns. Don’t go near guns. If you see a gun, run away and get an adult.” Others felt children should be instructed on how to use guns safely: “the four safety rules are: You treat every weapon as is loaded. The second one is: You never point your weapon at anything you do not intend to shoot. The third safety rule is: You keep your finger straight and off the trigger until you’re ready to fire, and you keep your weapon on safe until you’re ready to fire.” Many saw normalizing firearms as important to safety: “You can’t keep children unexposed from firearms because what happens? They get curious, and that’s when accidents happen.” Some participants even used toy guns or BB guns as tools for teaching children about gun safety. While most participants described teaching their children about firearm safety at home, two participants described enrolling their children in a firearm safety course at the local gun range. Several reported the belief that education for children was important because safer firearm storage does not completely prevent child access: “There’s no such thing as a perfect physical barrier. Everything is breach-able.” Six participants cited the mandatory Connecticut State Pistol Permit course as important in providing this information (State Pistol Permit 2022).

Some participants endorsed state firearm regulations for injury prevention. Participants discussed both background checks and Connecticut’s child access prevention law, which holds individuals legally liable if a child accesses an unlocked firearm and injures themselves or others (Azad et al. 2020). Four participants stated that this law influenced their storage practices.

Additional factors contributed to the parental conceptualization of firearm injury prevention. Several participants did not tell their children about the presence or location of firearms in the home. Two participants cited the belief that their child was unlikely to be injured due to firearm characteristics, including the safety mechanism, and the firearm is too large or cumbersome for a child to use. Some asked other parents if they owned firearms before their children could visit. Regional differences in firearm ownership and firearm culture were described. For example, one participant stated “People think it’s crazy that I’m getting a gun or people look down on it and they don’t do that in Texas or the Midwest.”

**Fig. 1:**
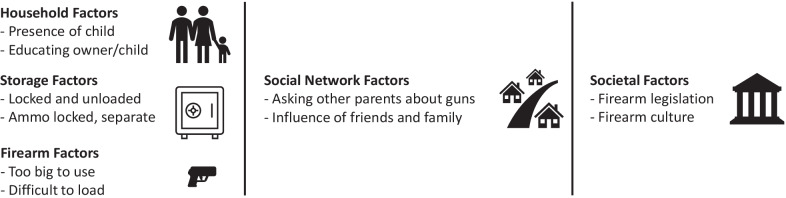
Social–ecological model of firearm safety among parents with firearms. A conceptual representation of perceived determinants of firearm safety among parents who keep firearms organized in accordance with the social–ecological model

Based on these findings, firearm injury prevention may be influenced by factors acting at different levels—some acting at the level of the individual, some within a social network, and others within a society. The social–ecological model (SEM) is a conceptual framework for organizing factors that affect health outcomes at the individual, relationship, community, and societal levels (The Social-Ecological and Model 2022). The SEM has previously been applied to firearm injury prevention (Durkin et al. 2020), but not specifically in the pediatric population. Figure 1 presents perceived determinants of firearm safety with respect to children identified in the study organized in accordance with the social–ecological model.

## Discussion

We sought to further characterize why parents engage in different firearm storage behaviors, what sources parents use to inform decisions about firearm storage, and identify barriers to using safer storage practices. We identified the following five themes: (1) firearm storage must be compatible with a specific context of use; (2) some parents engage in higher-risk storage because they believe it is adequate to reduce the risk of injury; (3) firearm storage practices are influenced by one’s social network and lived experience; (4) parents who own firearms may be amenable to changing storage practices; (5) parents’ conceptualization of firearm injury prevention is multimodal involving storage, education of owners and children, and legislation.

Our observation that a perceived need to access firearms quickly for self-defense may contribute to higher-risk storage practices is consistent with previous research (Aitken et al. 2020). Some individuals may be amenable to using safes and lockboxes specifically designed to facilitate rapid (Aitken et al. 2020; Simonetti et al. 2019). We identified a subset of parents with firearms who engage in higher-risk storage because they believe it is adequate to reduce the risk of injury. Some participants expressed confidence that their children had the necessary education to not injure themselves with firearms. Further research may clarify if this is due to optimism bias, where individuals perceive their own risk as less than that of others (Costa-Font et al. 2009). Interestingly, the risk of intentional self-injury was rarely mentioned by participants, despite accounting for a substantially greater number of firearm deaths than unintentional injury (WISQARS^TM^ 2022).

Firearm storage practices were commonly influenced by the practices of family and friends. This finding is consistent with what has been referred to as a social gun culture (Kalesan et al. 2016). Future research should explore how social networks can be leveraged to promote safer storage.

Many participants had either previously improved the safety of their firearm storage or were open to improving firearm storage practices. Participants who had changed storage practices often did so soon after having a child, suggesting that new parents may be especially open to adopting safer storage practices. Additional investigation on the impact of early counseling by pediatricians on parental firearm storage practices is needed.

Parents’ conceptualization of firearm injury prevention involved involving storage, education, legislation, and other practices. Most participants engaged in at least one of the safer storage practices associated with reduced risk of childhood firearm injury. Nearly all parents/guardians universally endorsed the importance of firearm safety education for parents and children. However, several studies have found that educational interventions, such as the NRA Eddie Eagle program, do not prevent children from handling firearms (Kalesan et al. 2016; Hardy et al. 1996; Hardy 2002; Himle et al. 2004). While only a minority of participants explicitly cited firearm regulation as important in preventing child injury, child access prevention laws have been shown to protect against both suicide and unintentional injury (Ando et al. 2014). Some participants cited firearm-related factors such as size or complexity of use; however, these are inadequate given that children as young as two years of age have discharged firearms and caused harm to themselves or others (Joliet shooting 2022). Finally, we presented a conceptual framework for perceived determinants of firearm injury prevention among parents who keep firearms in Fig. 1. We posit that the social–ecological model may provide a useful framework for understanding firearm safety in households with children. Our findings support that parental decisions around firearm storage and beliefs about firearm injury prevention are complex and unique to the individual. Counseling to improve firearm storage must therefore seek to understand parental beliefs, acknowledge individual autonomy, and enhance parents’ own motivation to change to accomplish the shared goal of preventing child injury. Experts in firearm injury prevention have advocated for a harm reduction approach in counseling interventions (Beidas et al. 2020), and brief motivational interviewing has been shown to be effective in improving firearm storage (Barkin et al. 2008).

There are several limitations to this study. Most participants were white, had high income, and were college-educated. This sample is not representative of New Haven County, where 23% of residents are non-white, the median household income is $70,000, and 35% of residents are college-educated (QuickFacts New Haven County 2022). Therefore, these findings may not reflect the perspectives of other sociodemographic groups and may not be generalizable. Firearm-related legislation and firearm practices vary regionally and may have a variable impact on firearm storage. Our study findings are specific to individuals residing in Connecticut. Finally, while safer firearm storage reduces the risk of suicide and unintentional injury, it has not been demonstrated to reduce firearm assault, which is an important contributor to child and adolescent morbidity and mortality and disproportionately affects youth of color (Cunningham et al. 2018).

## Conclusions

Parents who keep firearms value convenience and utility, which may be at odds with safer storage practices. However, some may be open to adopting these practices, especially after having a child. Family and peer relationships, education, and legislation represent important facilitators of firearm storage practices. Understanding these attitudes and beliefs on firearm storage may inform future interventions to improve storage practices.

## Data Availability

De-identified datasets generated during and/or analyzed during the current study are available from the corresponding author on reasonable request.

## Abbreviations

CS: Christopher Schenck
MW: Meghan Wilson
GT: Gunjan Tiyyagura
KB: Kirsten Bechtel
